# Anticoagulants inhibit proteolytic clearance of plasma amyloid beta

**DOI:** 10.18632/oncotarget.23718

**Published:** 2017-12-27

**Authors:** Lu Yang, Arup Bhattacharya, Yun Li, Yuesheng Zhang

**Affiliations:** ^1^ Department of Pharmacology and Therapeutics, Roswell Park Cancer Institute, Buffalo, NY, USA; ^2^ Department of Urology, Roswell Park Cancer Institute, Buffalo, NY, USA; ^3^ Department of Cancer Prevention and Control, Roswell Park Cancer Institute, Buffalo, NY, USA

**Keywords:** Alzheimer’s disease, amyloid beta, amyloid beta degradation, anticoagulant, Gerotarget

## Abstract

We recently discovered a plasma proteolysis pathway, termed the FXII-FVII pathway which is composed of coagulation proteases, and found it to be mainly responsible for the clearance of Aβ42 in the plasma in mice. Aβ42 and Aβ40 are the main Aβ forms in Alzheimer’s disease (AD). In the present study, *in vitro* assays, wild type (WT) mice and J20 mice (a transgenic AD model) are used to assess the degradation of Aβ40 and Aβ42 by the FXII-FVII pathway and the impact of anticoagulants on such degradation. Four clinically available and mechanistically distinct anticoagulants are evaluated, including dabigatran, enoxaparin (EP), rivaroxaban and warfarin. Each anticoagulant significantly elevates plasma level of synthetic Aβ42 in WT mice, among which EP is the most effective. The differential efficacies of the anticoagulants in elevating plasma Aβ42 level match closely with their inhibitory mechanisms towards the FXII-FVII pathway. Plasma Aβ40 is also degraded by the FXII-FVII pathway and is protected by EP. Moreover, the FXII-FVII pathway is significantly activated in J20 mice, but EP inhibits the activation and significantly elevates plasma levels of both Aβ40 and Aβ42. Taken together, our results shed new light on Aβ metabolism, reveal a novel function of anticoagulants, and suggest a novel approach to potentially developing plasma Aβ as an AD biomarker.

## INTRODUCTION

Amyloid beta (Aβ) is the major component of senile plaque and is widely considered a key driver of Alzheimer’s disease (AD) [1, 2]. Animal studies strongly suggest that targeting brain Aβ is an important therapeutic strategy against AD [3–5]. However, in clinical trials Aβ-targeted therapies have been ineffective [6, 7]. The negative clinical results raised the question of whether AD is triggered mainly by Aβ build-up in the brain, but it is also widely argued that Aβ-directed therapies should be given before significant brain damage occurs [8, 9]. Unfortunately, early detection of AD remains a major clinical challenge.

Different Aβ isoforms are generated from the Aβ precursor protein (APP), but Aβ40 and Aβ42 are the main isoforms [10, 11]. *APP* gene mutation or increased gene dosage (Down syndrome) causes increased Aβ production in the brain, leading to Aβ accumulation in the brain and increased Aβ release to peripheral circulation and cerebrospinal fluid (CSF) [12, 13]. Neuronal expression of mutated human APP in mice also increases Aβ production, causes AD-like disease, and increases Aβ level in the blood and CSF [14, 15]. Both decreased Aβ clearance and increased Aβ production in the brain have also been reported in sporadic AD [16, 17]. However, whereas plasma Aβ level can increase up to 2–3 fold in familial AD patients and Down syndrome patients [12, 13], it has a poor association with sporadic AD, which comprises approximately 95% of AD [18, 19]. Plasma Aβ cannot currently serve as an AD biomarker.

**Figure 1:**
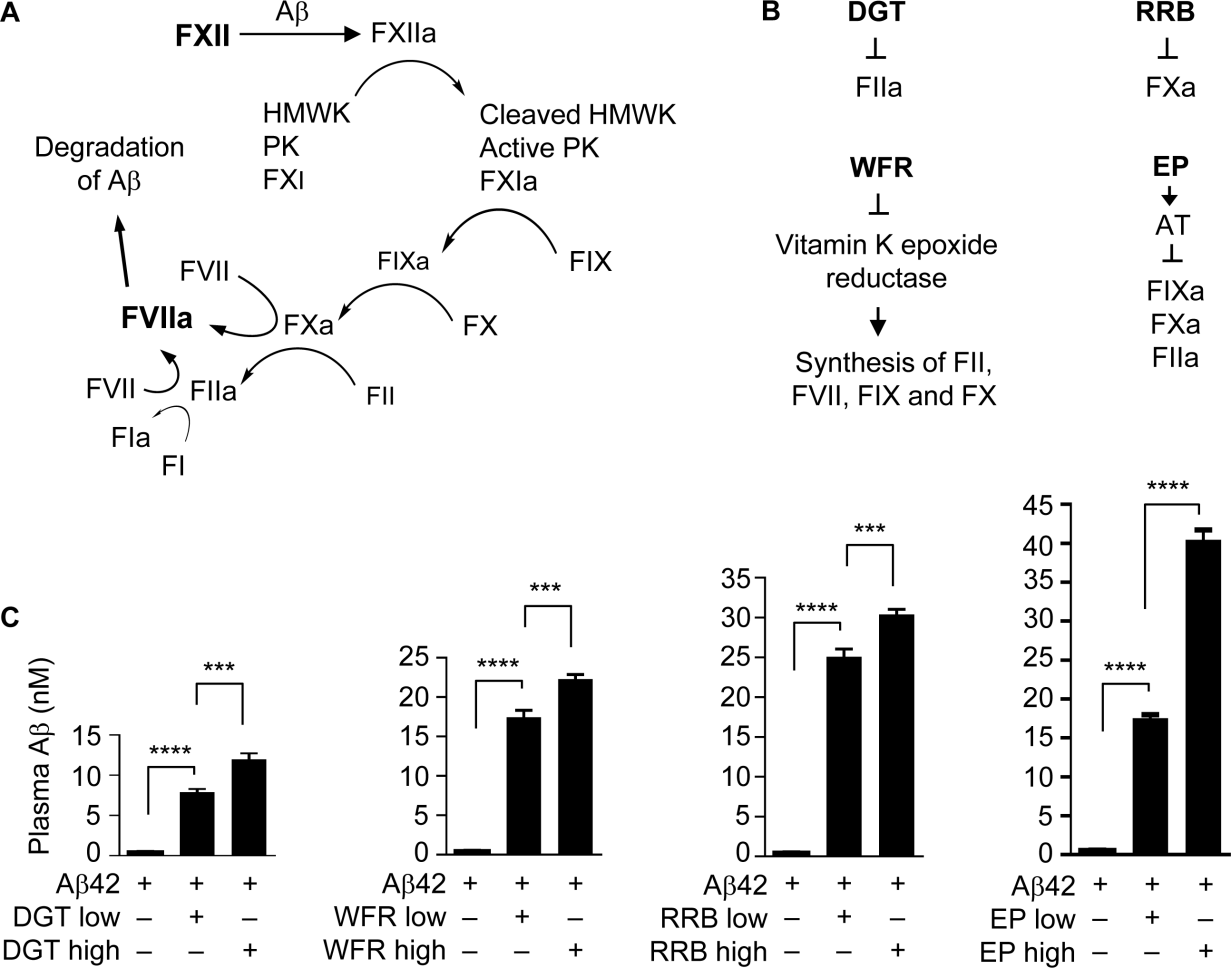
Anticoagulants elevate plasma Aβ42 level (**A**) The FXII-FVII proteolysis pathway that detects and degrades Aβ. (**B**) Coagulation factors that are inhibited by each anticoagulant. (**C**) Male C57BL/6 mice were treated with an anticoagulant or vehicle once daily for 5 days; 1 h after the final treatment, the mice were given a single dose of Aβ42 (40 μg/kg) and 6 h later blood was drawn from the mice for measurement of plasma Aβ concentration. The doses of the anticoagulants are as follows (mg/kg): DGT at 22.5 (low) and 45 (high), WFR at 1 (low) and 3 (high), RRB at 10 (low) and 20 (high), and EP at 0.1 and 0.5. DGT, WFR and RRB were given to mice p.o., whereas EP and Aβ42 were dosed i.p.. Each value is mean ± SD (*n =* 3); ^***^P < 0.001; ^****^P < 0.0001.

We recently found that Aβ42 is degraded in the plasma. Plasma Aβ42 binds and activates coagulation factor XII (FXII), which causes the activation of factors in the intrinsic and common coagulation pathways, including high molecular weight kininogen (HMWK), prekallikrein (PK), XI (FXI), IX (FIX), X (FX), II (FII; prothrombin) and I (FI; fibrinogen); activated FX and FII each activate factor VII (FVII; a key component of the extrinsic coagulation cascade); and activated FVII (FVIIa) degrades Aβ42 (Figure 1A) [20]. Other investigators previously showed activation of FXII and several downstream coagulation factors by Aβ but did not link it to activation of FVII and Aβ degradation by FVIIa [21, 22]. We also found that enoxaparin (EP), a low molecular weight heparin, which inhibits FIXa, FXa and FIIa (the activated forms of FIX, FX and FII) by activating antithrombin III [23, 24], disrupts the FXII-FVII pathway and elevates plasma level of exogenously administered Aβ42 in mice [20]. We found that about 60% of plasma Aβ generated endogenously is degraded by the FXII-FVII pathway under normal conditions [20]. These findings suggest that the FXII-FVII pathway may play an important role in Aβ clearance in AD. They also suggest that the poor association of plasma Aβ level with sporadic AD, mentioned above, may be related to its degradation in the plasma and that pharmacologic inhibition of the degradation pathway may reveal that plasma Aβ level is significantly higher in patients with AD or under AD development than in healthy individuals.

In the present study, we compared four clinical anticoagulants, which are mechanistically distinct, for inhibition of degradation of plasma Aβ42 in mice, examined the impact of the FXII-FVII pathway and an anticoagulant on plasma Aβ40, and investigated the status of the FXII-FVII pathway in a transgenic mouse model of AD and the effect of an anticoagulant on plasma Aβ level in these mice. The study was intended to gain better understanding about plasma Aβ degradation by the FXII-FVII pathway and the impact of anticoagulants on plasma Aβ levels.

## RESULTS

### Various anticoagulants elevate plasma Aβ42 level

We compared the inhibitory activities of four anticoagulants, including rivaroxaban (RRB, a specific FXa inhibitor) [25], dabigatran (DGT, a specific FIIa inhibitor) [26], warfarin (WFR, inhibiting the synthesis of vitamin K-dependent factors, including FII, FVII, FIX and FX) [27], and EP, which inhibits FIXa, FXa and FIIa as mentioned before (see also Figure 1B). C57BL/6 mice were treated with each agent or vehicle orally (p.o.) or intraperitoneally (i.p.) once a day for 5 days, and 1 h after the final dose, the mice were given Aβ42 at 40 μg/kg i.p., followed by blood drawing 6 h later. Notably, the multi-day treatment with an anticoagulant was based on our previous observation that at least three daily treatments of EP were needed to achieve maximal inhibition of the FXII-FVII pathway [20]. Mice were treated with DGT at 22.5 and 45 mg/kg, RRB at 10 and 20 mg/kg, and WFR at 1 and 3 mg/kg, which were deemed to render strong inhibition of their targets [28–30]. Mice were treated with EP at 0.1 and 0.5 mg/kg, based on our recent observation. Each anticoagulant markedly and dose-dependently elevated plasma level of Aβ42; EP was the most effective (up to 73.3 fold increase), whereas DGT was the least effective (up to 21.3 fold increase) (Figure 1C). The extent to which EP at 0.5 mg/kg elevates plasma Aβ42 level is nearly identical to that achieved with EP at 2.5 mg/kg [20], indicating maximal effect of EP.

### Degradation of plasma Aβ40 by the FXII-FVII pathway and its inhibition by EP

**Figure 2:**
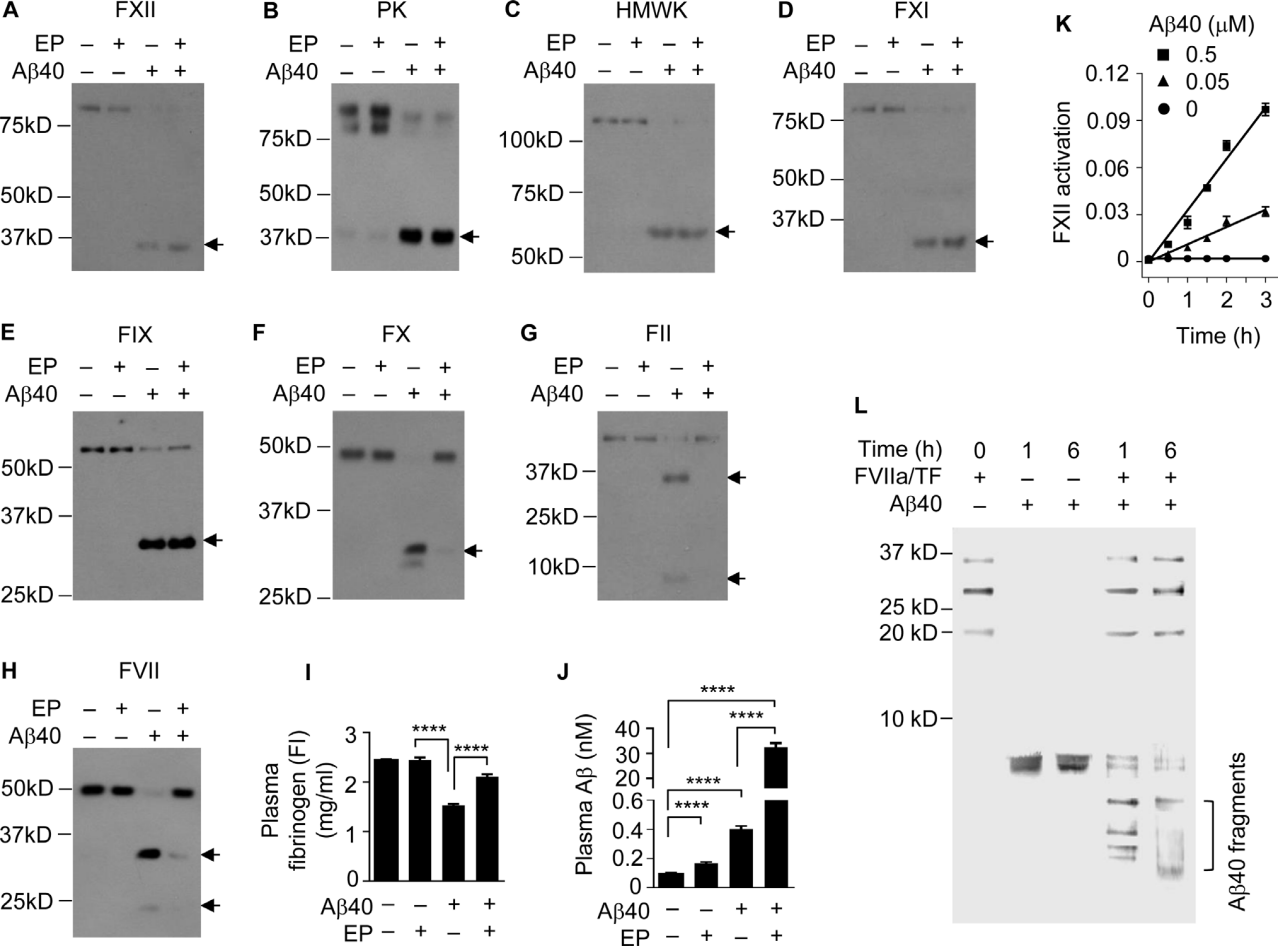
The FXII-FVII pathway detects and degrades Aβ40 (**A–J**) Changes in plasma coagulation factors in male C57BL/6 mice treated with EP and/or Aβ40. Mice were treated with vehicle or EP (0.5 mg/kg) i.p. once daily for 5 days, and 1 h after the final treatment, the mice were injected i.p. with vehicle or Aβ40 (40 μg/kg). Blood samples were obtained from the mice at 6 h after Aβ40/vehicle treatment; 7.5 μl plasma per sample was analyzed by Western blotting. Arrows indicate the cleaved fragments of the coagulation factors. Plasma levels of FI and Aβ were measured by ELISA. Error bars in I and J indicate SD (*n =* 3); ^****^P < 0.0001. (**K**) Aβ40 (0, 0.05 and 0.5 µM) was incubated with FXII (0.97 nM) in ZnCl_2_-containing PBS at RT; FXII activation was measured by a chromogenic assay. Each value is mean ± SD (*n =* 3). (L) Aβ40 (2.2 µM) was incubated alone or with FVIIa (10 nM) plus TF (10 nM) in CaCl_2_-containing PBS at RT for indicated times, and an equal volume of each incubation was separated by SDS-PAGE and stained by silver. FVIIa and TF were incubated without Aβ40, as a control.

Aβ40 differs from Aβ42 by only two amino acids and was previously shown to bind to FXII [31]. To test whether Aβ40 is also degraded by the FXII-FVII pathway, we gave C57BL/6 mice a single dose of human Aβ40 (40 μg/kg) or vehicle i.p. and 6 h later drew blood from the mice. Other mice were given EP (0.5 mg/kg) or vehicle i.p. once daily for 5 days, and 1 h after the last EP/vehicle dose, Aβ40 (40 μg/kg) was injected to the mice i.p., followed by blood drawing 6 h later. Analysis of plasma coagulation factors by Western blotting showed that Aβ40 activates the FXII-FVII pathway, activating FXII, PK, HMWK, FXI, FIX, FX, FII, FVII and FI, and that EP prevents the activation of FX, FII, FVII and FI (Figure 2A–2I), which closely resembles the changes in mice treated with Aβ42 with or without EP [20]. EP elevated plasma level of endogenous Aβ by 1.7 fold but increased plasma level of exogenously administered Aβ40 by 77.4 fold (Figure 2J), which is also similar to the effect of EP on plasma level of Aβ42 injected into the mice (Figure 1C). We confirmed that Aβ40 activates FXII and is degraded by FVIIa (Figure 2K and 2L). FVIIa is a trypsin-like serine protease, cleaving peptide bonds at the carboxyl side of arginine and lysine [32]. The cleavage pattern of Aβ40 suggests that FVIIa likely cuts Aβ40 at all three sites where an arginine (residue #5) or a lysine (residues #16 and 28) exists.

### Activation of the FXII-FVII pathway in AD transgenic mice

**Figure 3:**
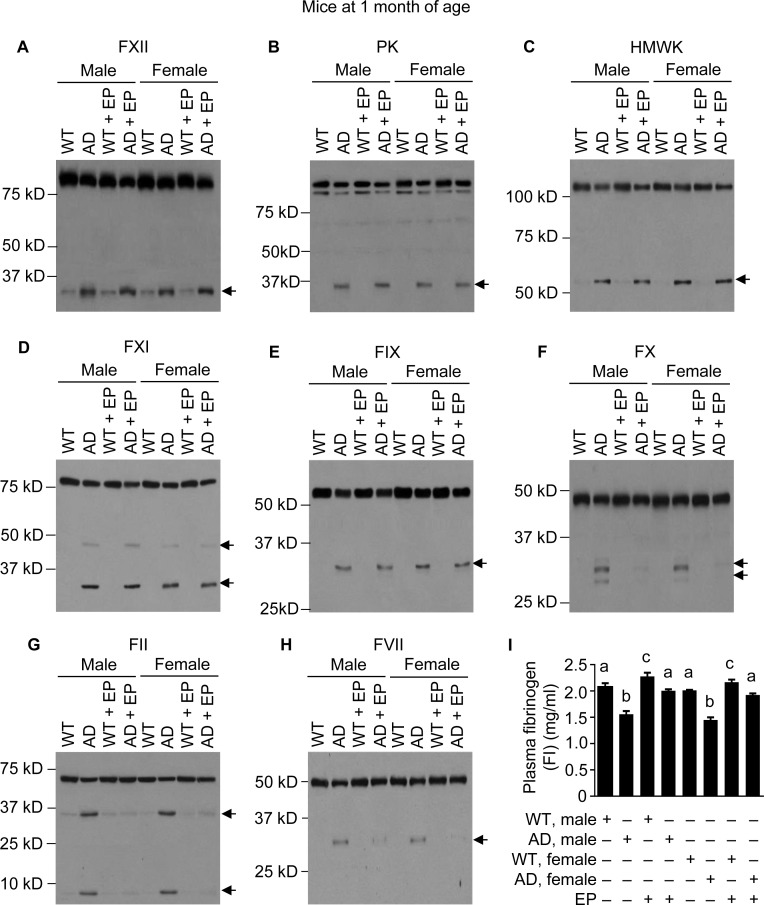
FXII-FVII pathway activation in J20 mice at 1 month of age, and the inhibitory effect of EP J20 mice and their age-matched WT littermates were treated with vehicle or EP (0.5 mg/kg) i.p. once daily for 5 days; 6 h after the final treatment, blood samples were obtained from the mice for measurement of coagulation factors by Western blotting (7.5 μl per sample) (**A–H**) and by ELISA (**I**). Arrows in (A–H) indicate cleaved fragments. Each value in I is mean ± SD (*n =* 3), and values annotated by different alphabetical letters are statistically different from one another.

**Figure 4:**
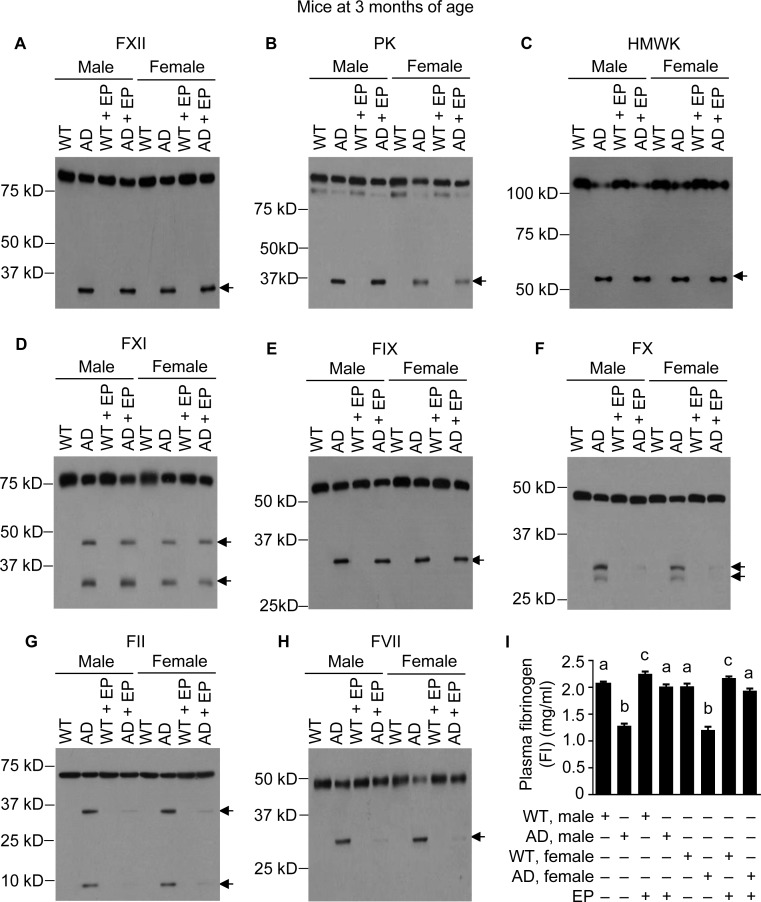
FXII-FVII pathway activation in J20 mice at 3 months of age, and the inhibitory effect of EP J20 mice and their age-matched WT littermates were treated with vehicle or EP (0.5 mg/kg) i.p. once daily for 5 days; 6 h after the final treatment, blood samples were obtained from the mice for measurement of coagulation factors by Western blotting (7.5 μl per sample) (**A–H**) and by ELISA (**I**). Arrows in (A–H) indicate cleaved fragments. Each value in I is mean ± SD (*n =* 3), and values annotated by different alphabetical letters are statistically different from one another.

**Figure 5:**
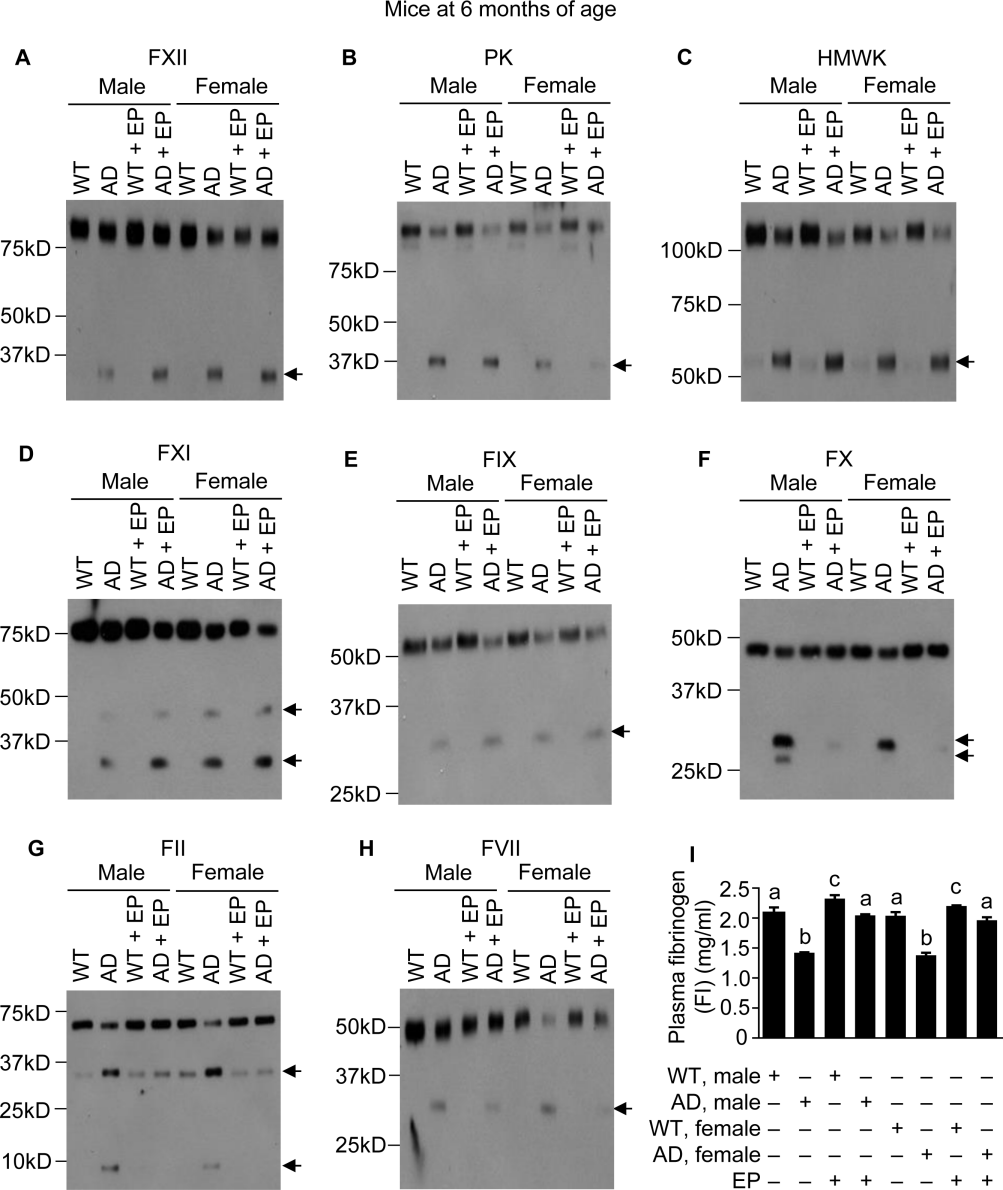
FXII-FVII pathway activation in J20 mice at 6 months of age, and the inhibitory effect of EP J20 mice and their age-matched littermates were treated with vehicle or EP (0.5 mg/kg) i.p. once daily for 5 days; 6 h after the final treatment, blood samples were obtained from the mice for measurement of coagulation factors by Western blotting (7.5 μl per sample) (**A–H**) and by ELISA (**I**). Arrows in (A–H) indicate cleaved fragments. Each value in I is mean ± SD (*n =* 3), and values annotated by different alphabetical letters are statistically different from one another.

Since administrating either Aβ40 or Aβ42 to mice activates the FXII-FVII pathway, and EP and other anticoagulants disrupt the activation, we hypothesized that the FXII-FVII pathway is activated in Aβ-generating AD transgenic mice and that EP elevates plasma Aβ level in these mice. We tested this hypothesis in J20 mice, which express in neurons human APP bearing both the Swedish (K670N/M671L) and the Indiana (V717F) mutations (APPSwInd) [33]. We analyzed J20 mice and WT littermates at age of 1, 3 and 6 months. At 1 month of age, the FXII-FVII pathway is already significantly activated in both male and female J20 mice, as shown by the activation of FXII, PK, HMWK, FXI, FIX, FX, FII, FVII and FI (Figure 3A–3I). EP greatly attenuated the activation of FX, FII, FVII and FI in these mice (Figure 3F–3I). In the WT littermates, only slight activation was detected of certain factors (FXII, HMWK and FII), and EP only slightly elevated FI level, which is consistent with our previous observation that the proteolysis pathway is only slightly activated under normal conditions [20]. At age of 3 and 6 months, activation of the FXII-FVII pathway in J20 mice is more pronounced, as shown by the increasing intensity of the cleaved form(s) of each factor and further decrease in FI level (Figures 4A–4I and 5A–5I), relative to that at 1 month of age. EP again strongly inhibited the activation of FX, FII, FVII and FI (Figures 4F–4I and 5F–5I). The activity of the FXII-FVII pathway remains low in the age-matched WT littermates; background cleavage of these factors are hardly visible due to short film exposure times, but EP does slightly elevate FI level (Figures 4 and 5).

### Significant increase in plasma Aβ level in EP-treated J20 mice

**Figure 6:**
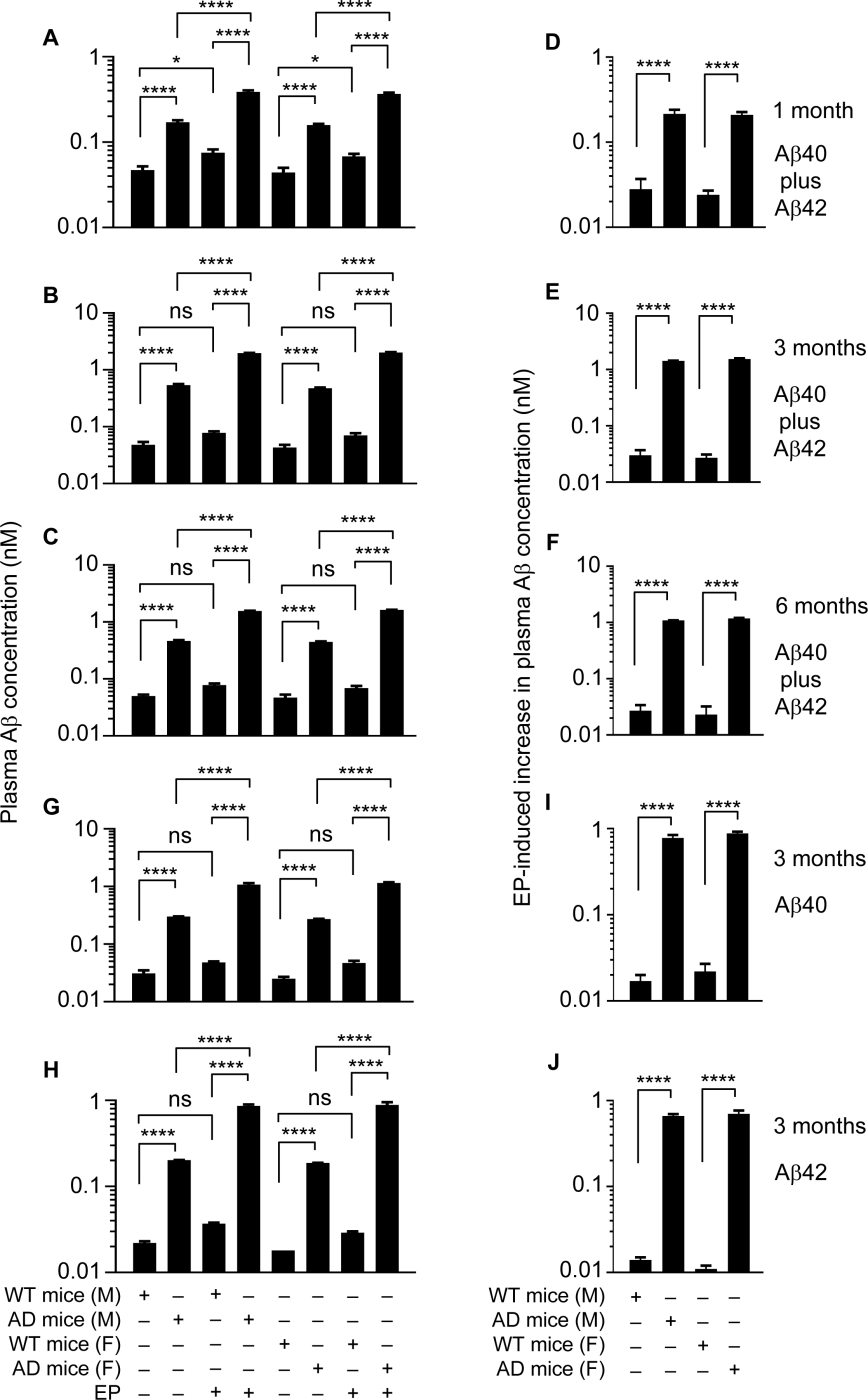
EP effect on plasma Aβ level in J20 mice and WT littermates J20 mice and their age-matched WT littermates were treated with vehicle or EP (0.5 mg/kg) i.p. once daily for 5 days; 6 h after the final treatment, blood samples were obtained from the mice for measurement of plasma Aβ concentration by ELISA. Each value is mean ± SD (*n =* 3). ^****^p < 0.0001; ns, not significant.

We first used an ELISA which measures both Aβ40 and Aβ42, as both Aβ species are present in the plasma of J20 mice [34]. In the WT littermates, plasma Aβ concentration is approximately 45.5 pM, and EP elevates it by approximately 1.6 fold, regardless of age or gender (Figure 6A–6C). In J20 mice, at 1 month of age, plasma Aβ concentration is 3.6 fold (male or female) higher than in the WT littermates, reflecting increased neuronal Aβ production in these mice; however, plasma Aβ concentration increased another 2.3 fold (male or female) in J20 mice after EP treatment (Figure 6A). The extent of EP-induced increase in plasma Aβ level suggests that more than 56% of plasma Aβ is degraded by the FXII-FVII pathway in J20 mice. Plasma Aβ level in J20 mice at 3 months of age is 2.8–3.0 fold higher than at 1 month age, reflecting progressive increase in neuronal Aβ production. At 3 months of age, without EP treatment, plasma Aβ level is 11.1–11.3 (female-male) fold higher in J20 mice than in the WT littermates (45.5 pM); plasma Aβ level increased another 3.7–4.2 fold (male-female) in J20 mice after EP treatment (Figure 6B). The extent of increase in plasma Aβ level induced by EP suggests that more than 73% of plasma Aβ is degraded by the FXII-FVII pathway in J20 mice. Plasma Aβ level appears to plateau at 3 months of age, as its level at 6 months of age is 94–98% (female-male) of that at 3 months of age (Figure 6C). Nevertheless, at 6 months of age, plasma Aβ level is 9.3–9.5 fold (male-female) higher in J20 mice than in the WT littermates (48.5 pM) without EP treatment; plasma Aβ level increased another 3.4–3.6 fold (male-female) in J20 mice after EP treatment (Figure 6C). The extent of increase in plasma Aβ level induced by EP at 6 months of age suggests that more than 70% of plasma Aβ is degraded by the FXII-FVII pathway in J20 mice.

Perhaps a more useful measure of the impact of EP on plasma Aβ level is the ratio of EP-induced increase in plasma Aβ level in J20 mice over that in the WT littermates. The ratio is 9.3 (male) and 8.7 (female) at 1 month of age, 47.5 (male) and 56.9 (female) at 3 months of age, and 40.4 (male) and 51.2 (female) at 6 months of age (Figure 6D–6F), which shows the striking effect of EP on plasma Aβ level in J20 mice.

We also analyzed plasma samples from mice at 3 months of age using ELISA kits specific for Aβ40 or Aβ42. In J20 mice, average plasma levels of Aβ40 are 271.0–300.7 pM (female-male), which are 10.8–9.7 fold higher than in the WT littermates (Figure 6G), and average plasma levels of Aβ42 are 186.7–202.3 pM (female-male), which are 10.4–9.2 fold higher than in the WT littermates (Figure 6H). Plasma levels of Aβ40 and Aβ42 increased 3.6–4.3 and 4.3–4.7 fold (male-female), respectively, in J20 mice after EP treatment but increased only 1.6–1.9 and 1.7–1.6 fold, respectively, in the WT littermates (Figure 6G and 6H). The ratio of EP-induced increase in plasma Aβ level in J20 mice over that in the WT littermates is 45.7 (Aβ40, male), 40.1 (Aβ40, female), 47.3 (Aβ42, male), and 63.6 (Aβ42, female) (Figure 6I and 6J). Thus, EP has similar effect on plasma levels of Aβ40 and Aβ42 in the mice.

## DISCUSSION

The multi-step and multi-component nature of the FXII-FVII pathway (Figure 1A) implies that many clinically available anticoagulants may disrupt it. Indeed, all four anticoagulants evaluated strongly increase plasma level of exogenously administered Aβ42 in mice (Figure 1C), and the differential efficacies of the anticoagulants conform to their mechanisms of action against the FXII-FVII pathway (Figure 1B). Aβ40 is also degraded by the FXII-FVII pathway and is protected by EP (Figure 2). The FXII-FVII pathway is significantly activated in J20 mice (Figures 3–5), which is consistent with increased release of brain Aβ to blood circulation in these mice. As expected, EP disrupts the FXII-FVII pathway (Figures 3–5) and strongly elevates plasma levels of both Aβ40 and Aβ42 in these mice (Figure 6).

The present data together with our recent work [20] show that the FXII-FVII pathway detects and degrades Aβ40 and Aβ42 in the plasma. Soluble low-density lipoprotein receptor-related protein-1 (sLRP) binds 70% of Aβ40 and 90% of Aβ42 in the plasma [35]. Since our data show that at least 56–73% of plasma Aβ is degraded by the FXII-FVII pathway in J20 mice, this pathway likely degrades both free and sLRP-bound Aβ. The impact of EP on plasma Aβ level is more dramatic in WT mice that received a bolus injection of Aβ than in J20 mice. This is likely due to heightened activation of the FXII-FVII pathway resulting from rapid entry of a large amount of Aβ into the blood circulation. Our results extend previous findings showing activation of FXII and certain downstream coagulation factors by Aβ [21, 22] by demonstrating that such activation leads to activation of FVII and Aβ degradation by FVIIa. The FXII-FVII pathway may conceivably impact AD pathogenesis in two opposite directions: 1) contributing to AD pathogenesis by promoting coagulation and inflammation, and 2) protecting against AD by degrading plasma Aβ. However, it remains controversial as to whether removing peripheral Aβ reduces brain Aβ level and slows AD development. For example, peripheral Aβ clearance via administration of Aβ binding protein gelsolin is therapeutically active in Tg2576 AD transgenic mice [5], but enhanced peripheral Aβ degradation via administration of neprilysin to APP23 mice and Tg2576 mice did not impact brain Aβ level [36, 37].

Other mechanisms of Aβ clearance include but not limited to Aβ internalization by astrocytes, Aβ degradation by extracellular or intracellular proteases, peripheral Aβ sink such as sLRP, and apolipoprotein E-mediated Aβ clearance [38, 39]. Plasmin also degrades Aβ [40], but plasma plasmin is unlikely to contribute significantly to Aβ degradation [41]. The FXII-FVII pathway appears to be a major Aβ-degrading mechanism in the plasma.

By inhibiting the FXII-FVII pathway, anticoagulants present an interesting dichotomy in impacting AD: Inhibiting procoagulant activity, potentially slowing or ameliorating AD, but also inhibiting plasma Aβ degradation, potentially contributing to brain Aβ accumulation. Indeed, increased coagulation is implicated in AD [42], but there is also evidence that peripheral Aβ moves to the brain [43]. The exact effect of anticoagulants on AD remains unclear. Treatment of AD mice (APP23 mice) with EP (∼2.5 mg/kg, i.p., 3 times weekly) for 6 months reduced brain astrocyte activation and Aβ accumulation [44], but chronic treatment of Tg2576 mice with EP at the same dose regimen significantly increased brain amyloid plaque load [45]. A recent human study has shown that chronic use of oral anticoagulants is associated with reduced risk of dementia in patients with atrial fibrillation [46], suggesting that these agents may slow AD development. Aβ degradation by the FXII-FVII pathway and its inhibition by anticoagulants represent a new cardiovascular variable that may potentially impact AD development. Notably, it was recently reported that AD patients have greater variability of both systolic and diastolic blood pressure [47, 48].

Although our present data were generated in preclinical models and J20 mice have higher plasma Aβ level than do AD patients, FXII activation by Aβ has been shown in AD patients [21, 22, 49]. Therefore, it is likely that in AD patients and those at presymptomatic or prodromal AD, the FXII-FVII pathway is significantly activated and degrades plasma Aβ. The FXII-FVII status in these patients should be examined. Our data also suggest that anticoagulants may elevate plasma Aβ level more significantly in patients with increased Aβ production than healthy individuals. Since anticoagulants are clinically available, it seems feasible to address the above question in a clinical setting. Such studies may show that even a short-term use of an anticoagulant may significantly increase plasma Aβ level in these patients due to inhibition of the FXII-FVII pathway. Notably, anticoagulants are widely used to treat atrial fibrillation and venous thromboembolism and in patients with mechanical heart valves [50–52]. It is also conceivable that by transiently inhibiting the FXII-FVII pathway, an anticoagulant may allow plasma Aβ to better reflect Aβ synthesis in patients and may potentially convert plasma Aβ into an AD biomarker, facilitating early detection and enabling early treatment of the disease.

## MATERIALS AND METHODS

### Reagents

DGT and EP were purchased from Combi-Blocks and Fresenius Kabi, respectively. RRB and WFR were purchased from Advanced ChemBlocks and Bristol-Myers Squibb, respectively. Human Aβ40, human Aβ42, soybean oil and Solutol HS 15 were purchased from Sigma-Aldrich. FXII, FVIIa and tissue factor (TF) were purchased from Haematologic Technologies. Antibodies for HMWK and FII were purchased from Santa Cruz Biotechnology. Antibodies for FXII, PK, FXI, FIX, FX and FVII were purchased from GeneTex.

### Mouse study

All experiments were approved by the Institutional Animal Care and Use Committee at the Roswell Park Cancer Institute under protocol 1022M. All mice were housed in individually ventilated pathogen-free cage systems equipped with HEPA filtered air supply and reverse osmosis purified water in a centralized vivarium facility. The facility is controlled with fully automated sensors for light (12-hour dark – 12-hour light cycle), temperature (70°F ± 2°F), humidity and airflow. All mice had free access to standard rodent diet (sterilizable Teklad #2018, with 6% fat and alfalfa free). C57BL/6NTac mice (male) were purchased from Taconic and used in two experiments. In the first experiment, mice at 8–9 weeks of age were treated with vehicle or an anticoagulant once daily for 5 days, and 1 h after the final treatment, the mice were treated with a single dose of Aβ42, followed by blood draw 6 h later. Blood was collected from the mice by cardiac puncture at the time of sacrifice by carbon dioxide and was collected into K3 EDTA-containing tubes (Multivette 600 from Sarstedt), from which plasma samples were prepared. DGT was dissolved in soybean oil containing 2% dimethyl sulfoxide. RRB was dissolved in 10% ethanol, 40% Solutol HS 15 and 50% water. WFR was dissolved in water. EP and Aβ42 were dissolved in phosphate-buffered saline (PBS). DGT, RRB and WFR were administered to mice p.o., whereas EP and Aβ42 were given to mice i.p.. In the second experiment, mice at 8–9 weeks of age were treated with vehicle or EP i.p. once daily for 5 days, and 1 h after the final treatment, the mice were treated with vehicle or Aβ40 i.p., followed by blood draw 6 h later. Aβ40 was dissolved in PBS. Each agent was administered to the mice in 0.1 ml volume per 20 g body weight each time.

J20 mice were purchased from the Mutant Mouse Resource & Research Centers (MMRRC) via the Jackson Laboratory. Hemizygous male J20 mice in C57BL/6J background were bred with WT female mice (C57BL/6J, also from Jackson Laboratory). F1 hemizygous J20 mice were identified by PCR genotyping based on the Jackson Laboratory protocol (https://www/jax.org/strain/006293) and used for experiments. J20 mice and their WT littermates (at the age of 1, 3 and 6 months, male and female) were treated with EP or vehicle i.p. once daily for 5 days, and at 6 h after the last treatment, blood was drawn from the mice.

### Measurement of plasma Aβ and FI

Plasma concentrations of Aβ were determined by ELISA, which either detects both Aβ40 and Aβ42 [53] or specifically measures each peptide. In the latter case, ELISA kits from Invitrogen (KHB3481 and KHB3441) were used. Plasma concentration of FI was also measured by ELISA using a kit from GenWay Biotech. The commercial kits were used by following the manufacturers’ instructions.

### Western blotting

Each plasma sample (7.5 μl of plasma per sample) was mixed with 4x loading dye, heated at 95°C for 5 min, and resolved by SDS-PAGE. The separated proteins were transferred to polyvinylidene fluoride membrane, probed with specific antibodies, and detected using Luminata Classico or Luminata Cresendo (Millipore).

### Measurement of FXII activation by Aβ40 and Aβ40 degradation by FVIIa

FXII activation by Aβ40 was measured by a chromogenic assay [22], monitoring the conversion of the chromogenic substrate for 3 h at room temperature (RT) using a microplate reader. To measure its degradation by FVIIa, Aβ40 was incubated with or without FVIIa plus cofactor TF in PBS in a total volume of 100 μl containing 5 mM CaCl_2_ for 1 or 6 h at RT. TF was solubilized in 10 mM CHAPS, which was diluted 10-fold in the final assay. FVIIa and TF were also incubated without Aβ40, as a control. Each sample at an equal volume was subjected to SDS-PAGE and stained by silver using a kit from Invitrogen (LC6070).

### Statistical analysis

Data were analyzed by analysis of variance (ANOVA), followed by Tukey multiple comparisons test using GraphPad Prism 7. For data that were highly skewed, log transformation was performed before ANOVA. Using the standard α = 0.05 cutoff, *p* < 0.05 was considered statistically significant. Group size of 3 mice was used in each experiment, which was deemed adequate for detecting the effects of an anticoagulant on the main endpoint (plasma Aβ level) based on pilot experiments. In the pilot experiments, we measured the variation of plasma Aβ level among WT mice and J20 mice and concluded that 3 mice per group are adequate for detection of 2-fold increase in plasma Aβ levels in an anticoagulant treatment group with at least 90% power. Our previous study in mice showed that disrupting the FXII-FVII pathway elevates plasma level of Aβ42 by at least 2 fold [20].
